# Engineering Biomimetic Sporangia in Land Plants

**DOI:** 10.3390/bioengineering13091003

**Published:** 2026-08-28

**Authors:** Matthew S. Grasso, Rachael Floreani, Philip M. Lintilhac

**Affiliations:** 1 Department of Plant Biology, The University of Vermont, Burlington, VT 05405, USA; mattsgrasso@gmail.com; 2 Department of Mechanical Engineering, The University of Vermont, Burlington, VT 05405, USA; floreani@uvm.edu

**Keywords:** plant reproduction, sporangia, differentiation, microfluidics, hydrogel, cytoskeleton, alginate-methacrylate, protoplast, gametogenesis

## Abstract

The land plant sporangium is unique in its ability to initiate differentiation of the reproductive germ line, leading directly to meiosis and the formation of eggs and sperm; but our lack of understanding of the processes underlying gametogenesis in plants remains a major obstacle in our ability to modify the sexual cycle for the benefit of crop science. Here we report an initial attempt to create an engineered structure which, with further development, could reproduce the physical and mechanical properties of the land plant sporangium. While the work reported here does not achieve the ultimate goal of in vitro gametogenesis, we do believe it represents a significant step towards understanding the stress-mechanical environment of the land plant sporangium and its role in initiating reproductive development. The immediate goal of this work is to extend the range of experimental methods and tools available for the study of the physical environment of plant cell growth. We describe an experimental protocol based on the microfluidic encapsulation of living plant protoplasts in multilayered microbeads composed of actively tunable hydrogel materials whose properties can be modified to mimic the stress-mechanical behaviors of the living sporangium. Our results demonstrate the first successful encapsulation of a living plant protoplast in dual-layered microbeads consisting of an inner layer of gelled agarose and an outer layer of crosslinked alginate/methacrylate.

## 1. Introduction

In the animal kingdom eggs and sperm differentiate early in life and are maintained as distinct lineages that persist through sexual maturity. This aspect of sexual development allows for the harvesting of naïve eggs and sperm for in vitro fertilization. But in land plants there is no comparable lineage of reproductive cells that is maintained during vegetative growth. Instead, reproductive cells differentiate de novo as the plant enters its reproductive phase. The de novo origin of the reproductive germ line in plants raises a fundamental question about the mechanisms underlying the shift in developmental fate to reproductive growth, meiosis, and gametogenesis. What is the inducing stimulus that locates and initiates archesporial differentiation in plants?

Recently, it has been proposed that the land plant sporangium evolved as a stress-mechanical focusing device that targets a precisely located region in the growing sporangium [1], thereby shifting the developmental fate of the most centrally located cell, or cells (the archesporium), towards reproductive differentiation and gametogenesis. In this work we take first steps towards reconstructing the physical architecture of the sporangium in an engineered structure. Previous work has shown that living plant protoplasts can be successfully encapsulated in hydrogel microspheres and maintained in vitro for extended periods of time [2]. Here we introduce a more advanced embedment methodology, showing that we can build multi-layered hydrogel microbeads comprising an inner agarose layer and an outer alginate–methacrylate (ALGMA) layer akin to a cortex and epidermis structure, suggesting that further development may open the possibility of constructing biomimetic sporangial structures that are capable of initiating archesporial differentiation and meiosis in vitro.

## 2. Experimental Approaches

While the goal of in vitro gametogenesis in plants is unattainable at present, the work reported here describes an experimental protocol designed to physically constrain individual plant cells in ways that allow us to manipulate stress, strain, and shear at the level of the individual cell while maintaining the ability to visualize cellular responses at the sub-cellular level.

Increasing interest in the use of microfluidic methodologies for the study of cell behavior [3] and cytoskeletal responses to cell constraint [4] have shown that plant cells are highly responsive to their mechanical environments and their constraint geometries [5].

All plant cells and tissues are equipped with mechanosensitive structures capable of reacting rapidly and precisely to changing load conditions [6,7,8]. The cellular structures that most likely fill this need are an assemblage of cytoplasmic proteins collectively referred to as the cytoskeleton, consisting of semi-rigid compressive rods known as microtubules, and tensile contractile fibers called microfilaments. The cytoskeleton, augmented by a number of accessory proteins, can together be regarded as a unified tensegrity structure [9,10] that is capable of reading stress, strain, and shear information at any location in the cytoplasm [11].

The hypothesis underlying this effort proposes that in the pre-meiotic sporangium, the specification and subsequent channeling of developmental fate towards reproductive differentiation and meiosis depends upon deterministic physical and mechanical information networks that have overcome many of the limitations of stochastic molecular information systems [12].

In the present study we apply single-cell microfluidic methods to one of the major outstanding questions regarding the origin of the germ line in land plants. Microfluidic methods have been applied to plant materials in the context of whole-organ or pollen tube responses to physical constraint [13]. Future advances in active hydrogel engineering should make it possible to control the shrinkage and/or swelling of the individual layers [14,15], to create localized isotropic singularities [1] capable of transmitting unique physical cues to individual cells at precise locations in a larger cell mass.

## 3. Materials and Methods

**Terminology**:

Protoplast: Living plant cells from which the cellulosic cell wall has been removed.

Microdroplet: Protoplasts encapsulated in ungelled agarose droplets.

Microsphere: Microdroplets that have been cooled to their gelling temperature.

Microbeads: Microspheres that have been re-encapsulated in a second layer of crosslinked hydrogel (alginate–methacrylate).

**Hydrogel Polymers:**

1. **Agarose:** Low-gelling-temperature Agarose #A9045 was obtained from Sigma-Aldrich Inc. Burlington, MA, USA.
2. **(AlgMA) Alginate–Methacrylate:** Lyophilized AlgMA polymer was provided by the Floreani Lab in the Dept. of Mechanical Engineering, University of Vermont, Burlington, VT, USA.

**Solutions and Culture Media:**

**(CWDS) Cell Wall Digestion Solution:** 9% mannitol, 0.05% MgCl2, 0.4% Cellulase RS, 0.4% Cellulase R-10, 0.05% Macerozyme R-10, 0.05% Pectolyase Y-23, 20 microliters Rohapect UF, 20 microliters Rohapect 10 L, 20 microliters protease inhibitor cocktail, 0.19% MES, and pH adjusted to 5.8 using KOH. The solution was sterilized using a rapid-flow Nalgene filter unit with a pore size of 0.43 µm.
**(PWS #1) Protoplast Washing Solution:** 9.3% mannitol, 0.05% MgCl_2_, 0.1% MES, and pH adjusted to 5.8.
**(PWS #2) Protoplast Washing Solution w/o MgCl_2_:** 9.3% mannitol, 0.1% MES, and pH adjusted to 5.8.
**Conditioned Medium** was collected from actively growing, 4-day-old *Nicotiana tabacum* cv. BY-2 suspension cultures by filtration through a rapid-flow 0.43-micron filter. Conditioned medium was stored in a freezer without autoclaving.
**(PCM) Protoplast Culture Medium:** 3% sucrose, 0.43% MS Medium (Caisson Labs, Smithfield, UT, USA), 0.2 mg/L 2,4-dichlorophenoxyacetic acid, 1 mg/L thiamine, 100 mg/L myo-inositol, 4.5% mannitol, 10 mL Conditioned Medium, and pH adjusted to 5.8 using 0.1 M KOH.
**(CBS) Cell Buoyancy Solution:** 0.5% sucrose, 1.65% high MW Dextran 150,000, 8.65% mannitol, 0.1% MES, 0.04% MgCl_2_, 4% (*v*/*v*) OptiPrep™ (Serumwerk, Bernburg, Germany), and pH adjusted to 5.8.
**(CS) Calcium Solution:** 4% mannitol, 1.46% CaCl_2_ 2H_2_O, and pH adjusted to 5.8 using 0.1 M KOH.
**(AMDS) Alginate–Methacrylate Droplet Solution:** 7.5% mannitol, 1% F68 Pluronic, 0.073% CaCl_2_ 2H_2_O (5 mM CaCl_2_), and pH adjusted to 5.8 using 0.1 M KOH.

* All prepared media were autoclave-sterilized unless otherwise noted.

**Step 1**—Protoplast isolation.

Although many sources of living plant protoplasts can be proposed, we began with an established plant cell culture line that is immortal and easily cultured to prepare wall-less protoplasts in quantity.

The *Nicotiana tabacum* BY-2 cell line (rpc00041) was provided by RIKEN BRC through the National BioResource Project of the MEXT, Tokyo, Japan. Protoplasts were obtained from 4-day suspension cultures grown at 27 °C in MS Medium (Murashige and Skoog basal salts, 3% sucrose, 100 mg/L myo-inositol, 1 mg/L thiamine, and 0.2 mg/L 2,4-dichlorophenoxyacetic acid).

Suspension-cultured cells were removed from the culture medium and enzymatically stripped of their cell walls in cell wall digestion solution (CWDS). Suspension-cultured BY-2 cells were digested for 2–4 h at 27 °C on a shaker table. The digestion was split into two parts. During the first part, BY-2 cells in digestion medium were placed in an incubating shaker (27 °C, 120 R.P.M.) for between 45 minutes and 1 h 20 minutes. The first part of the digestion was stopped when most cells had become spherical in shape but were still attached to their neighbors. They were then transferred to an incubator (27 °C, no shaking) for between 1 h 15 min and 2 h 40 min. The second step of the protoplast generation process was stopped when almost all protoplasts had been released from their neighbors. Protoplasts were separated from the digestion medium by centrifugation at 100× *g* for 7 minutes and washed twice in protoplast wash solution 1 (PWS #1). Protoplasts were then resuspended in a cell buoyancy solution (CBS) and diluted to a concentration of 2250 protoplasts/µL in preparation for the encapsulation process.

**Step 2**—Single-layer microsphere production.

**Figure 1:**
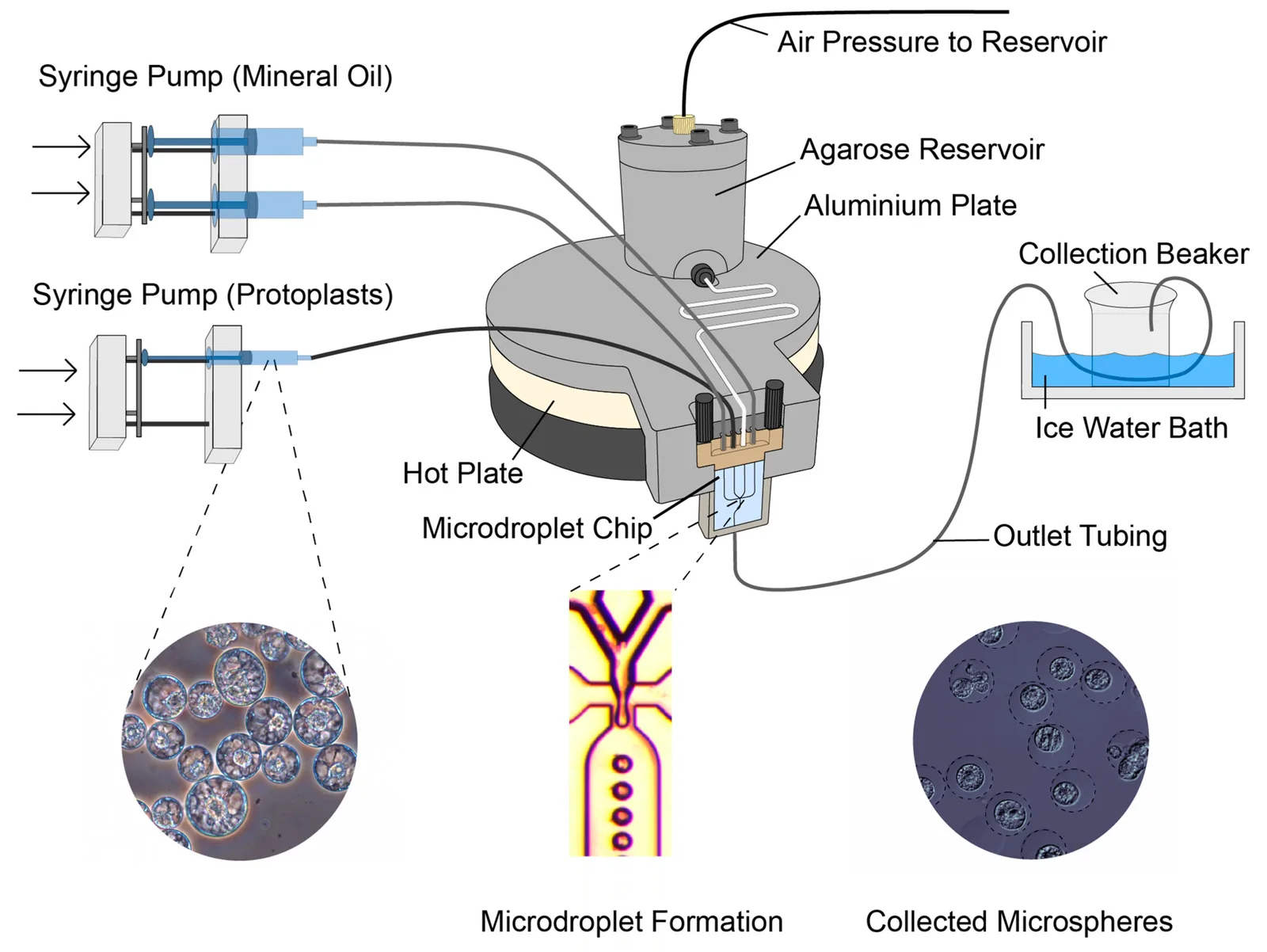
Agarose microsphere encapsulation system. Two separate syringe pumps control the flow of protoplasts and mineral oil into a Dolomite Microfluidics 4-channel, 2-reagent, hydrophobic microdroplet chip. Agarose flows from a pressurized and heated reservoir into the microdroplet chip, with input line temperatures maintained at 35 °C by a channeled aluminum plate and liquid agarose reservoir atop a digital hot plate. Outlet tubing carries liquid microdroplets in oil through an ice water bath that cools the agarose into solid microspheres before depositing them into a sterile collection beaker. Agarose microspheres taken from the collection beaker are washed and centrifuged.

Agarose microspheres were generated using a commercial microfluidic-droplet system (Dolomite Microfluidics; Blacktrace holdings, Royston, UK). Water-based (agarose) droplets were formed as the discontinuous phase in a 2-reagent, 4-channel, hydrophobic junction chip (Junction diameter 100 u). The continuous phase was a light mineral oil (Sigma Aldrich, St. Louis, MO, USA) with 2% Span 80 (Fluka Analytical, St. Louis, MO, USA) added to prevent droplet coalescence. Flow rates of the protoplasts and agarose were adjusted with separate Dolomite P-Pumps independently controlled by proprietary Dolomite software. The mineral oil continuous phase was controlled by a syringe pump. The fluids were fed into the droplet chip through 4 equal lengths of polytetrafluoroethylene (PTFE) microbore tubing (Figure 1).

Of the two central aqueous channels one was reserved for the protoplasts prepared in Step 1. The second central aqueous channel was fed from a warmed reservoir containing 1.5% low-gel-temperature agarose (Sigma-Aldrich) dissolved into PWS #1 and maintained at a temperature of 35 °C. Agarose solution was prefiltered through 0.22 µm syringe filters for sterilization.

**Figure 2:**
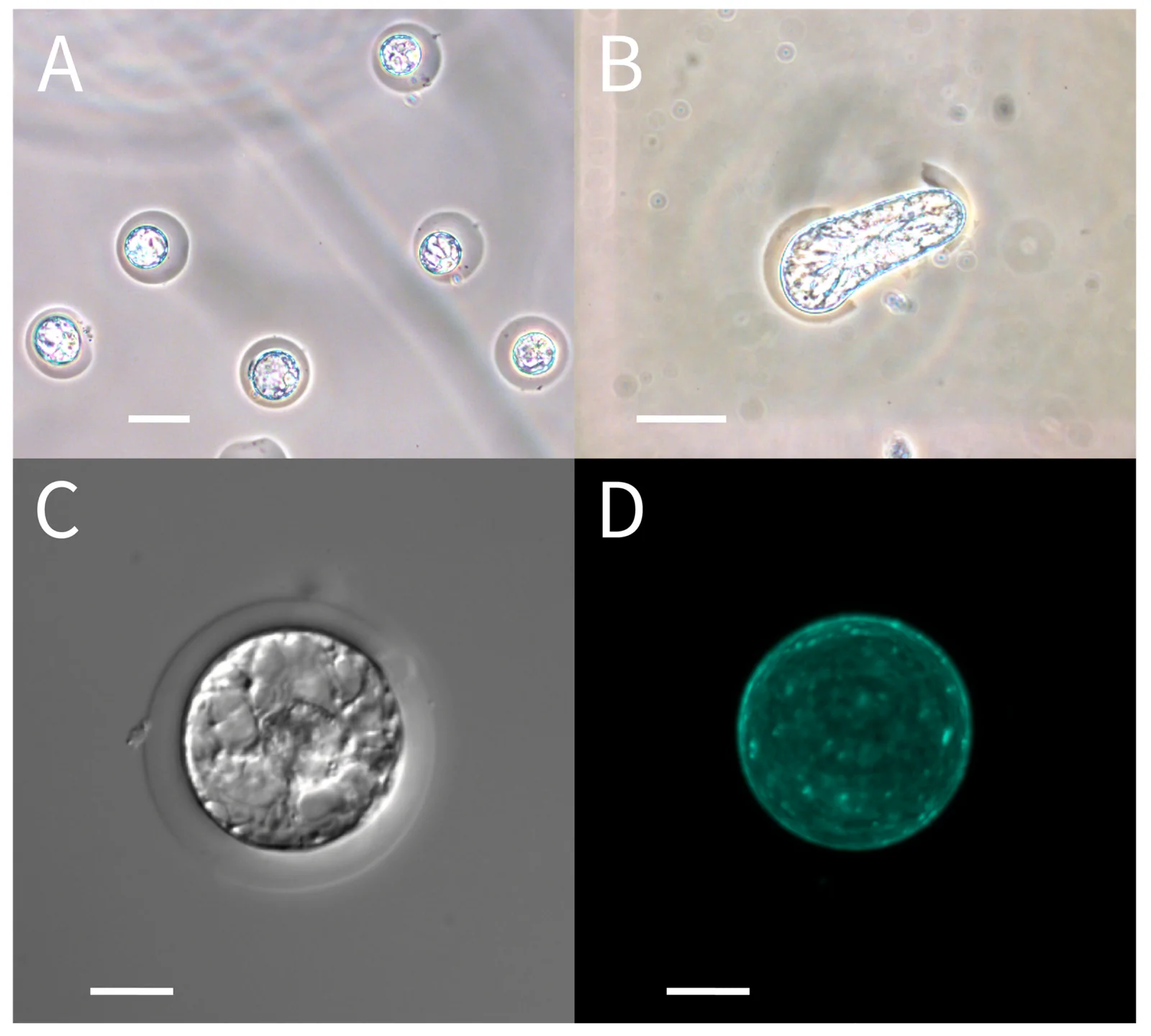
(**A**) Phase-contrast image of BY-2 protoplasts in solidified microspheres suspended in MS Medium immediately after encapsulation. (**B**) Phase-contrast image of a BY-2 cell rupturing the agarose microbead 6 days after encapsulation. (**C**) Nomarski differential interference contrast image of an individual BY-2 Cell 24 h after encapsulation showing active cytoplasmic streaming. (**D**) Calcofluor White fluorescence of the cell in (**C**) showing regeneration of a thin cell wall. Scale bars = 50 μm (**A**,**B**) and 20 μm (**C**,**D**).

With appropriate flow control, a stream of monodisperse agarose droplets was formed at the chip junction where the aqueous and oil phases intersect (Figure 1). Droplet diameter can be increased or decreased by adjusting the flow rates of the continuous and discontinuous phases. Liquid droplets exit the chip into outlet tubing, which carries them through an ice bath, causing microdroplets to solidify into microspheres before being deposited into a collection beaker (Figure 2).

Consistently sized (ca 60 µm) spherical hydrogel microspheres were successfully generated at a rate of approximately 130 droplets/sec. Individual protoplasts were successfully encapsulated with good viability as determined by cytoplasmic streaming activity shortly after encapsulation (Figure 2C). Cellulose staining with Calcofluor White showed that 24 h after initial protoplast release all living cells had regenerated a thin surrounding wall (Figure 2D). The amount of cell wall regenerated continued to increase up to 6 days after encapsulation in agarose microspheres. Living cells proceeded to elongate and divide, eventually bursting the agarose microsphere in which they had originally been encapsulated (Figure 2B). Immediately following droplet formation, approximately 25% of droplets contained protoplasts.

Agarose-encapsulated protoplasts in the mineral oil continuous phase were transferred from the collection beaker to a 15 mL conical centrifuge tube and 6 mL of PWS #1 was added. The tube was centrifuged for 4 min at 800 RPM, forming a pellet in the aqueous phase. The bottom of the tube was punctured with a sterile needle and microspheres were drained out and collected. This solution was filtered through a sterilized 149-micron nylon mesh to remove large pieces of debris. At this point microspheres were centrifuged again to form a pellet and then transferred to PCM. They were placed in an incubator set to 27 °C with no shaking.

**Figure 3:**
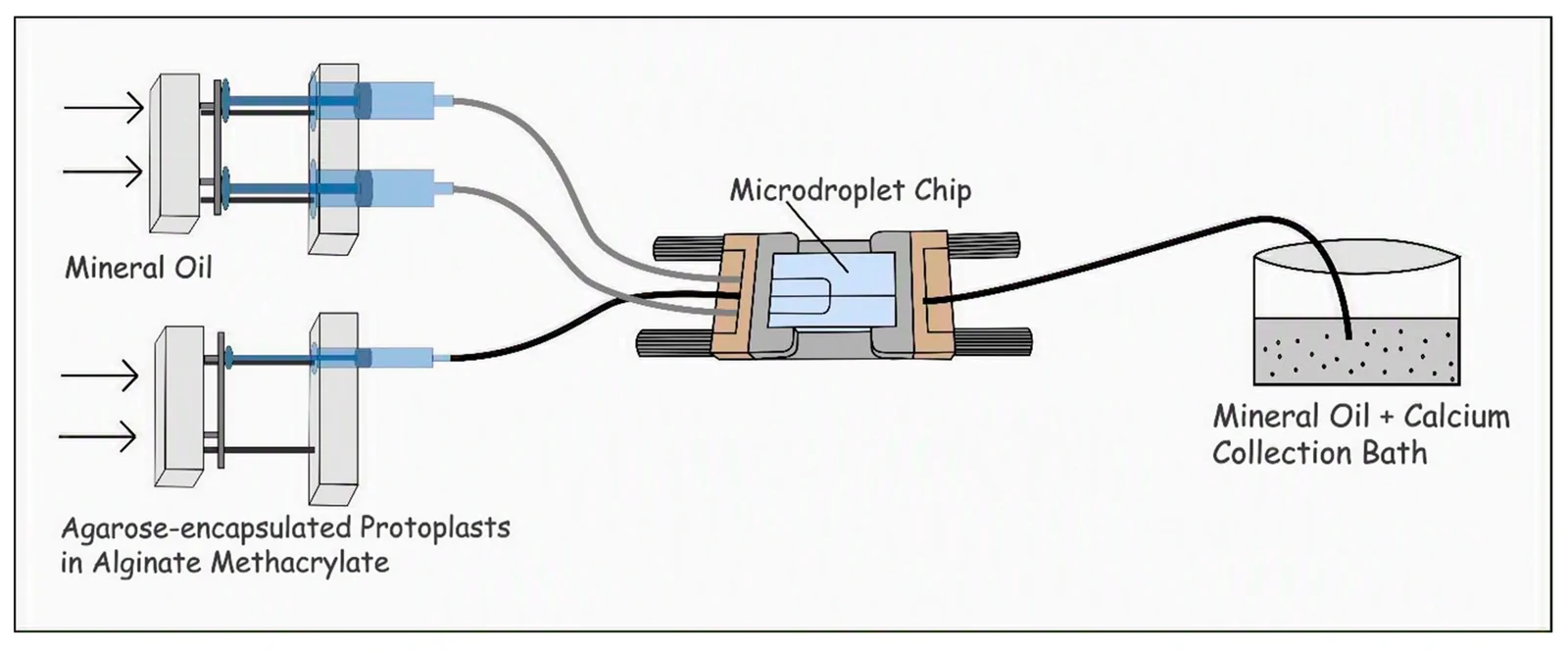
Agarose-encapsulated protoplasts are pre-mixed into an AlgMA stream flowing into faster-moving mineral oil, resulting in agarose microspheres encased in a layer of AlgMA.

**Step 3**—Dual-layer agarose and alginate–methacrylate microbead encapsulation of agarose microspheres (Figure 3).

Agarose-encapsulated protoplast microspheres were collected after 24 h of culture and centrifuged at 100× *g* in a 15 mL conical centrifuge tube. After the supernatant was decanted microspheres were re-suspended in protoplast washing solution PWS #2 and allowed to rest for 15 min to wash out any salts found in the PCM that might cause premature gelling of the alginate–methacrylate (AlgMA). The washed microspheres were centrifuged to form a pellet and re-suspended in PWS w/o MgCl_2_ to a final density of approximately 650 agarose microspheres/µL.

AlgMA solution was prepared by first sterilizing 0.02 g of dry AlgMA polymer by saturating it with 100% ethyl alcohol and allowing the alcohol to evaporate in a sterile cabinet. Once evaporated, the dry sterile polymer was dissolved in 0.7 mL of AMDS by shuttling the solution and polymer between two 3 mL syringes connected by a Luer couple for 30 min. Once fully dissolved, 300 µL of agarose microsphere-encapsulated protoplasts in PWS w/o MgCl_2_ were added to the AlgMA solution and mixed by slowly shuttling back and forth between the two syringes ~30 times. Encapsulation within agarose protects protoplasts from shearing forces caused during Step 3, which results in agarose microspheres being suspended in a liquid (ungelled) AlgMA carrier in anticipation of a final round of microfluidic-droplet encapsulation.

**Figure 4:**
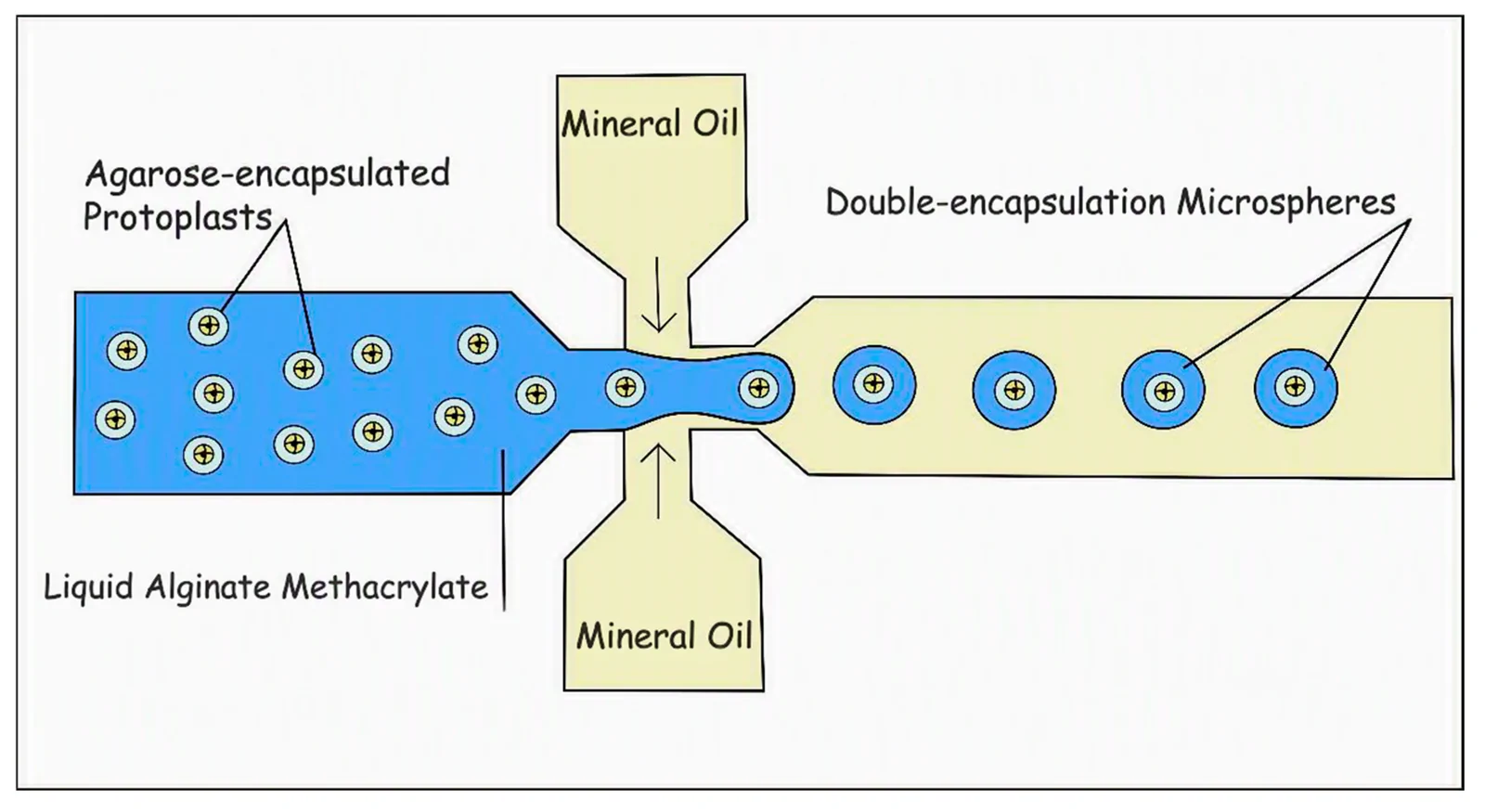
Schematic of the 3-channel microdroplet chip used for AlgMA double encapsulation.

**Step 4**—Dual-layer AlgMA microbead encapsulation protocol:

AlgMA microbead generation was done using a 3-channel, 190 µ junction diameter, hydrophobic, flow-focusing microdroplet chip from Dolomite Microfluidics (Figure 4). The continuous phase was mineral oil with 2% Span 80. The flows of the AlgMA solution and mineral oil were controlled separately by two syringe pumps (Figure 3 and Figure 4).

**Step 5**—Final collection and gelation of microbeads.

**Figure 5:**
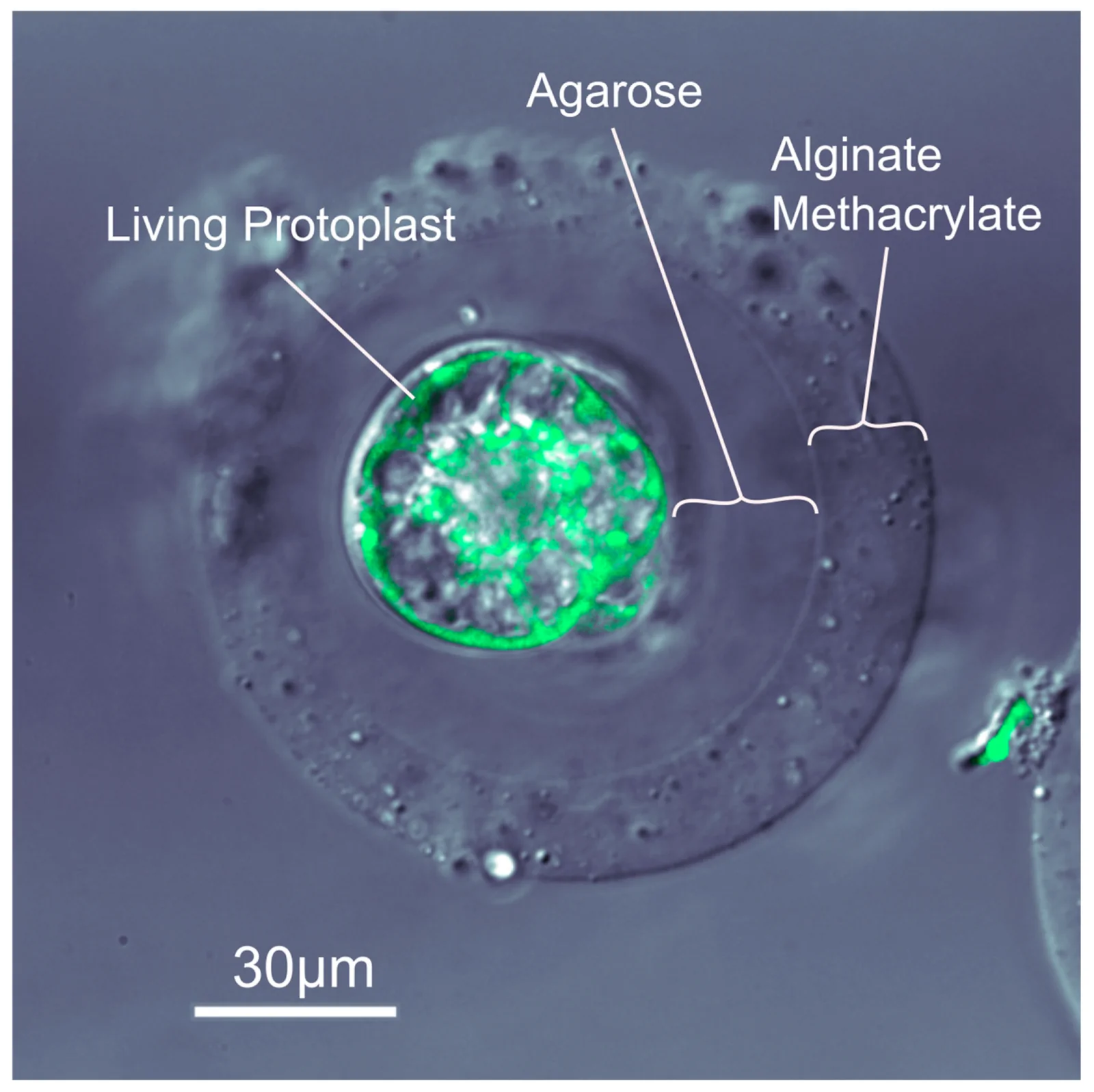
Overlay of a differential interference contrast and fluorescein diacetate stained fluorescence image of an AlgMA/agarose dual-layer microbead showing an actively streaming, living protoplast. Image was taken 3 days after protoplast isolation and 2 days after AlgMA encapsulation. The living protoplast is stained green with FDA viability stain. Labels show the outer layer of AlgMA and the inner layer of agarose that encapsulates the living protoplast.

For the final collection of microbeads, dry CaCl_2_ was added in powder form (10 mM Ca^2+^ equivalent) as an initial crosslinking agent to 5 mL of mineral oil and 2% span 80 and mixed with a magnetic stir bar for 2 h before final encapsulation. During encapsulation the CaCl_2_ mineral oil bath was continuously stirred (Figure 4). A schematic of the AlgMA encapsulation system is shown in Figure 4. The syringe pump controlling the flow of oil was set to 1000 µL/hour and the syringe pump controlling the flow of protoplasts in AlgMA was set to 150 µL/hour. The system was run for one hour. The collected calcium-crosslinked AlgMA microbeads in mineral oil (Figure 5) were added to a 15 mL conical centrifuge tube along with 6 mL Calcium Solution (CS) and centrifuged at 100 RPM for 4 min to transfer microbeads from the mineral oil layer to the CS. AlgMA microbeads were drained from the bottom of the tube and allowed to sit in the CS for 10 min to ensure complete calcium crosslinking. After 10 min, microspheres were pelleted again by centrifugation and the CS supernatant was removed and replaced with 3 mL of PCM. Dual-layer microspheres were transferred to a small Petri dish and placed in an incubator at 27 °C. Note that there are two stages to the calcium crosslinking, the first using powdered CaCl_2_ in oil, and the second using CaCl_2_ in aqueous solution. The use of AlgMA also enables further crosslinking to be done, modifying the stiffness of the gels using visible-light crosslinking systems [16].

## 4. Buoyancy Control

Precise control of protoplast buoyancy in the suspending medium is essential. Freshly prepared protoplasts, being denser than the suspending medium, will settle onto the lower, inner surface of the microbore feed lines where they will tend to clump. It is essential to keep them uniformly suspended in the carrier medium. We have found that a high-density, non-toxic radiological contrast agent such as Iodixanol (Fisher OptiPrep™) can be used to maintain neutral density at critical times during microdroplet and microbead assembly, as well as for controlling buoyancy during centrifugal phase separation. Iodixanol is non-toxic and does not significantly alter osmotic balance with changing concentration. It can be pH-adjusted and autoclave-sterilized without disrupting its properties.

## 5. Results

Single-layer protoplast/agarose microspheres were pre-mixed into the prepared AlgMA solution. Dual-layer AlgMA microbeads (Figure 5) were generated using a flow-focusing microfluidic chip and a continuous water-immiscible phase consisting of mineral oil with 2% Span 80. The calcium-oil collection bath crosslinked AlgMA droplets into consistently shaped microbeads. The crosslinked AlgMA microbeads were separated from the calcium-oil collection bath through centrifugation into aqueous medium. While viability of dual-layer microbead/protoplasts was low, staining with fluorescein diacetate revealed living plant cells within dual-layer AlgMA/agarose microspheres (Figure 5). The total elapsed time from initial protoplast release to microbead assessment was 3 days.

This work represents the first attempt to reconstruct the multi-layered geometry of the land plant reproductive sporangium using hydrogel materials. We were able to generate dual-layer AlgMA microbeads containing living plant protoplasts in small numbers, losing a proportion of viable protoplasts at each step along the way. However, we have successfully demonstrated that it is possible to generate multilayered microbeads encapsulating living plant cells that remained viable.

Previous work has shown that viable single-layer encapsulation of living plant protoplasts is possible [2]. We can now demonstrate that multi-layer encapsulation generating spherical dual-layer droplets, each containing a viable BY-2 protoplast, is also possible. In addition, we show that dual-layer microbeads can be formed using different polymer systems with distinct mechanical behaviors, and that the dimensions of the resulting microbeads can be maintained within the range of the native sporangial structures that they emulate.

## 6. Unresolved Issues

In recent work we have outlined the underlying biomechanical logic of target selection in the earliest stages of reproductive differentiation in land plants [1]. Here we address the question of how the mechanical stimuli that target precursors of the archesporium can be investigated experimentally. Our work does not address the question of sourcing different cell lines from intact plant tissues, nor does it attempt to address the question of developmental competence, which may be established independently through hormonal pathways [16].

## 7. Discussion

The work reported here is a preliminary study. We believe that with further development, the viability of multi-layer encapsulated plant cells will improve, leading to reliable methodologies for manipulating the mechanical environment of individual plant cells. An anticipated outcome of future work would be the ability to elicit the differentiation of supernumerary archesporia in vitro and the subsequent induction of meiosis using engineered biomimetic sporangia, which can be produced in large numbers, leading to the possibility of harvesting haploid pseudo-gametes for various purposes.

The list of tools available for tuning the mechanical properties of hydrogel materials is growing. Further development of the methacrylated alginate methodology may allow the microsphere’s outer layer to be photo-crosslinked by free-radical polymerization, making the mechanical stiffness of the microspheres adjustable post-encapsulation. This would also result in covalent bonding between alginate chains, which would then be protected from destabilization in physiological solutions. Future advances in hydrogel engineering and crosslinking methods [17,18] using mechanically active hydrogel polymers raises the possibility of actively manipulating the intensity and location of center-focused stress fields in order to generate isotropic singularities [1] acting on appropriately competent living plant cells at targeted locations, under conditions that mimic those of living plant sporangia.

Although genetically scripted molecular controls necessarily underlie all developmental processes, there are intrinsic limits to the ability of molecular signals to coordinate precise structural decisions at the transcellular level. Information flows based on molecular population changes are necessarily stochastic at the tissue level [12], whereas physical signals, being intrinsically deterministic and less prone to the dissipative tendencies introduced by diffusion, do not rely on molecular transport at all, and can be directed to carry out action at a distance instantly.

The production of physical shape and form in plants, such as leaves, stems, and roots, necessarily reflects the material properties of their structural components and the way they interact with the mechanical signals imposed by the surrounding cellular environment; but while the details of the mechanical stress environment and surface topography can be tuned at the molecular level by transcriptionally directed inputs, the deterministic physical interactions that drive architecture at the cellular level must be understood independently. In turgid and growing plant organs, including nascent sporangial structures, the physical forces released into the body of the growing sporangium reflect the growth potential of the constituent cells, but they also reflect the geometry and surface topography of the sporangial structure as a whole, providing a continuously changing landscape of structure-specific information which is seamlessly updated with the changing shape of the sporangium as it continues to grow [19].

## 8. Conclusions

Physical and mechanical inputs are increasingly being recognized as pivotal in the development of plant form and function. But while the role of cell and tissue mechanics is correctly seen against a background of molecular control systems, we are beginning to understand that in the plant kingdom, developmental systems have evolved within the constraints of immotile cells, apoplastic continuity, and pressure-driven growth. Under these constraints cell and tissue mechanics have evolved into robust information networks that can operate in environments where stochastic molecular information systems are ineffective. With rapid advances in the capabilities of microfluidic technologies, and with the introduction of novel hydrogel materials capable of actively manipulating the mechanical environment of individually encapsulated cells, microfluidic methods may be positioned to play a central role in moving the science of plant developmental biology into a new era of experimental possibility and theoretical synthesis.

While the report presented here falls short of the ultimate goal of initiating archesporial differentiation and meiosis in plant materials, we outline first steps towards the generation of active, multicellular structures that mimic the developmental architecture of the living plant sporangium, comprising a turgid epidermal cell layer surrounding a centrally located isotropic singularity, moving plant science closer to the ultimate goal of in vitro reproductive differentiation.

## Data Availability

The original contributions presented in this study are included in the article. Further inquiries can be directed to the corresponding author.
